# Artificial intelligence in fundus photography for type 2 diabetes: a scoping review of systemic biomarkers and multi-organ risk prediction

**DOI:** 10.3389/fdgth.2026.1768780

**Published:** 2026-04-22

**Authors:** Liting Huang, Hongyun Lu, Mingqi Yang, Yanyan Liu, Mini Han Wang, Kang Zhang

**Affiliations:** 1 Faculty of Medicine, Macau University of Science and Technology, Taipa, Macao SAR, China; 2 Department of Endocrinology and Metabolism, Zhuhai People’s Hospital (The Affiliated Hospital of Beijing Institute of Technology, Zhuhai Clinical Medical College of Jinan University), Zhuhai, China; 3 Guangdong Provincial Key Laboratory of Tumor Interventional Diagnosis and Treatment, Zhuhai People's Hospital (The Affiliated Hospital of Beijing Institute of Technology, Zhuhai Clinical Medical College of Jinan University), Zhuhai, China

**Keywords:** artificial intelligence, biomarkers, clinical translation, deep learning, diabetic retinopathy, multi-organ complications, personalized medicine, retinal fundus imaging

## Abstract

Type 2 diabetes mellitus (T2DM) is associated with multi-organ complications, including cardiovascular and renal disease. Fundus photography provides a non-invasive window into systemic microvascular health, and artificial intelligence (AI) has enabled extraction of retinal biomarkers for systemic risk prediction beyond diabetic retinopathy detection. We conducted a methodologically structured scoping review following PRISMA-ScR guidance to map AI applications in retinal imaging for multi-organ risk stratification in T2DM. Studies using machine learning or deep learning models to predict cardiovascular, renal, or cerebrovascular outcomes were identified and characterized. Rather than quantitative pooling, we examined modeling strategies, validation approaches, performance reporting, and translational readiness across heterogeneous study designs. AI models frequently demonstrated promising discrimination; however, substantial heterogeneity was observed in cohort size, outcome definitions, imaging modalities, and validation strategies. External validation was limited, calibration was inconsistently assessed, and subgroup analyses addressing fairness and device-related domain shift were rarely reported. Most studies emphasized discrimination metrics without comprehensive evaluation of clinical utility.Retinal AI shows potential for scalable systemic risk surveillance in T2DM, but rigorous external validation, standardized reporting, and prospective implementation studies are required to enable safe and equitable clinical translation.

## 1 Introduction

Diabetes has become an increasingly severe public health issue worldwide, and it is estimated to affect 536.6 million adults (20–79 years) in 2021, projected to rise to 783.2 million by 2045 (1), of which more than 90% of cases are type 2 diabetes mellitus (T2DM). This epidemic imposes a significant burden not only due to hyperglycemia itself but crucially through its devastating multi-organ complications, including cardiovascular disease (CVD), stroke, chronic kidney disease (CKD), and vision-threatening diabetic retinopathy (VTDR). Individuals with T2DM face substantially elevated risks of myocardial infarction, cerebrovascular accidents, congestive heart failure, end-stage renal disease, and all-cause mortality (2). However, predicting these complications early remains challenging with current clinical strategies. Reliance on traditional risk factors and laboratory biomarkers, such as blood pressure measurements for hypertension or albuminuria and estimated glomerular filtration rate (eGFR) for diabetic kidney disease (DKD), often requires invasive blood or urine sampling. Moreover, these indicators may lack sensitivity in the early stages of disease and can be influenced by physiological variability, such as white-coat effects or diurnal fluctuations (3, 4). Therefore, there is an urgent need to develop non-invasive and highly precise methods for predicting the risk of diabetic complications, in order to mitigate adverse outcomes associated with missed or delayed diagnoses (4, 5). Consequently, non-invasive biomarkers capable of reflecting early systemic pathophysiological changes has become a major priority in diabetes research and clinical risk stratification.

The retina, as an embryological extension of the central nervous system, offers direct visualization of the microvasculature *in vivo* (6). Retinal fundus image emerges as a uniquely powerful and non-invasive window into systemic vascular and metabolic health. Color fundus photography (CFP) is relatively cost-effective, widely deployed in screening programs (especially for diabetic retinopathy), and avoids the invasiveness associated with many current systemic risk assessments. Changes visible in the retina are increasingly recognized not merely as indicators of localized ocular disease but as sensitive biomarkers reflecting systemic micro- and macrovascular pathophysiology associated with diabetes and its complications (2, 4, 5, 7). These findings highlight the retina's potential as an integrated indicator of systemic disease risk, and underscore the need to develop analytical approaches for assessing the correlation between retinal fundus photographs and diabetic complications.

**Figure 1:**
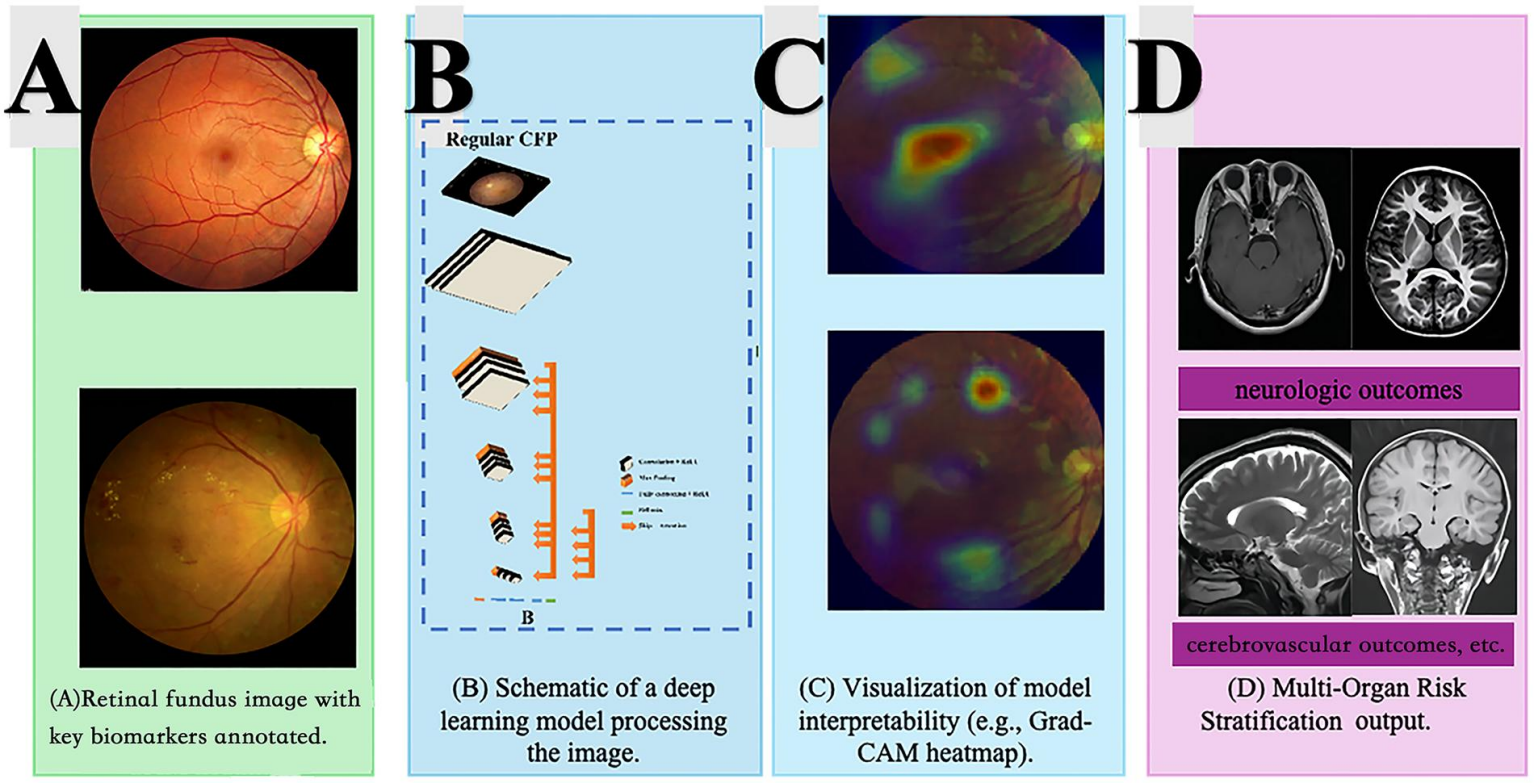
Overview of AI-based multi-organ risk prediction from retinal fundus imaging. **(A)** The representative color fundus photographs. Subpanel A-1 displays a retinal image with key ocular biomarkers (e.g., vessels, optic disc, macula) annotated. A-2 shows a Grad-CAM heatmap highlighting salient regions that drive the model's prediction, providing explainable-AI–based interpretability. **(B)** Schematic representation of the deep learning architecture that processes a standard color fundus photograph (CFP): the image undergoes feature extraction through successive convolutional layers and is mapped to risk-related representations. **(C)** Visualization model interpretability with additional Grad-CAM visualizations demonstrating localized regions of diagnostic importance. **(D)** The outputs to generate multi-organ risk stratification, linking ocular biomarkers to systemic endpoints such as cardiovascular and neurologic outcomes.

The advent of sophisticated Artificial Intelligence (AI), particularly deep learning (DL), has revolutionized the analysis of retinal images, unlocking their potential for systemic risk prediction beyond traditional ocular diagnostics. AI algorithms excel at detecting subtle, complex patterns and biomarkers in fundus images that may elude human observers or conventional methods (4–7). DL models have demonstrated strong performance (AUC 0.85–0.93) in identifying chronic kidney disease and type 2 diabetes from fundus images, alone or combined with clinical metadata, underscoring the retina's role as a window into systemic health (8). Multimodal deep learning (MMDL), which integrates retinal images with readily available clinical variables, has further shown superior performance for conditions like hypertension, DKD, and cardiovascular disease compared to unimodal models (3, 4, 7). Machine learning approaches, particularly tree-based learners and deep neural networks, frequently achieve higher sensitivity and specificity than traditional regression models for risk prediction and are adept at handling complex data like medical images (9). Globally, AI applications for predicting diabetic microvascular complications are expanding rapidly (10). While reviews report promising performance [e.g., a mean c-statistic of 0.81 for DKD prediction on internal validation (11)], there remains a need for more robust external validation, especially for complications beyond DKD. The field is evolving from single-modality analysis toward integrating retinal imaging with other data streams, such as electronic health records(EHRs) and genomic information, to develop more accurate and holistic predictive models (11). AI applied to routine retinal images moves beyond detecting diabetic retinopathy; it demonstrates significant potential (AUC range: 0.676–0.971 across complications) for opportunistic, non-invasive screening of multiple systemic complications, including DKD, cardiovascular disease, and neuropathy, offering a scalable solution for comprehensive risk prediction and early intervention (12). Figure 1 shows the overview of AI-based multi-organ risk prediction from CFP. The subfigures of Figure 1 are cited from Han's studies (13, 14).

This review summarizes current advances in AI applied to retinal imaging for systemic risk prediction in type 2 diabetes. We first outline the biological rationale for retinal biomarkers reflecting multi-organ microvascular and neurodegenerative processes. Next, we review retinal imaging modalities and AI methodologies, including traditional machine learning, deep learning, multimodal fusion, and explainable AI techniques. We then synthesize current evidence on AI models predicting cardiovascular, cerebrovascular, renal, and neurological outcomes. Finally, we discuss key challenges for clinical translation, including external validation, algorithmic fairness, interpretability, and regulatory considerations. Also,we highlight future research priorities for implementing retinal AI as a scalable, non-invasive platform for multi-organ risk surveillance in T2DM.

## 2 Methods

### 2.1 Study design

This study adopted a methodologically structured scoping review to evaluate and characterize the current evidence on AI applications in CFP for systemic risk prediction in T2DM. The review followed the Preferred Reporting Items for Systematic Reviews and Meta-Analyses Extension for Scoping Reviews (PRISMA-ScR) framework to ensure methodological transparency and reproducibility. A scoping review approach was selected because the field encompasses imaging modalities, AI architectures, and outcome definitions, which preclude formal quantitative synthesis. Therefore, this review aimed to identify and describe the breadth of existing studies, methodological trends, and translational readiness of AI-based retinal imaging approaches for predicting multi-organ complications associated with T2DM rather than to estimate pooled effect sizes.

### 2.2 Search strategy

A systematic literature search was performed across the following databases: PubMed, Embase, Web of Science, and IEEE Xplore. The search timeframe was set from January 1, 2017, to December 31, 2025. A combination of controlled vocabulary and free-text terms was used to ensure comprehensiveness and precision. Search terms were categorized as follows: (1) Retinal imaging: CFP, optical coherence tomography (OCT), optical coherence tomography angiography (OCTA); (2) Artificial intelligence: artificial intelligence, DL, ML; (3) Clinical context: T2DM, cardiovascular disease, kidney disease, systemic risk.

### 2.3 Eligibility criteria

Studies were included if they met the following criteria: research articles that applied machine learning or deep learning techniques to retinal imaging data for predicting systemic disease outcomes; studies that used retinal fundus images or related retinal imaging modalities as primary model inputs; studies involving individuals with T2DM or mixed populations in which systemic risk prediction relevant to diabetes complications was reported; and studies providing quantitative evaluation metrics such as area under the receiver operating characteristic curve (AUC) or related performance indicators. Studies were excluded if they were editorials, commentaries, case reports, conference abstracts without full methodological details, or non-English publications. Studies focusing solely on ophthalmic outcomes such as diabetic retinopathy detection without reporting systemic risk prediction were also excluded. This approach ensured that the review captured research directly linking retinal AI imaging with multi-organ risk stratification, aligning with the study's translational focus.

### 2.4 Study selection and data extraction

All retrieved records were independently screened by two investigators at the title/abstract stage and full-text stage; discrepancies were resolved through consensus. The study selection process was documented in a PRISMA-ScR flow diagram. A standardized data extraction form was used to collect key information, including: country, study population, sample size, imaging modality and acquisition details, model architecture, training strategy, validation scheme (internal split, cross-validation, external validation), and performance metrics (AUC, sensitivity, specificity). Calibration measures and subgroup analyses were also recorded. Given the substantial heterogeneity across studies in endpoint definition, cohort composition, modeling procedures, and reporting formats, a quantitative meta-analysis was not performed. Instead, a structured narrative synthesis was conducted to summarize methodological trends, evaluate the quality of validation, and analyze the translational potential for clinical implementation.

## 3 Results

**Figure 2:**
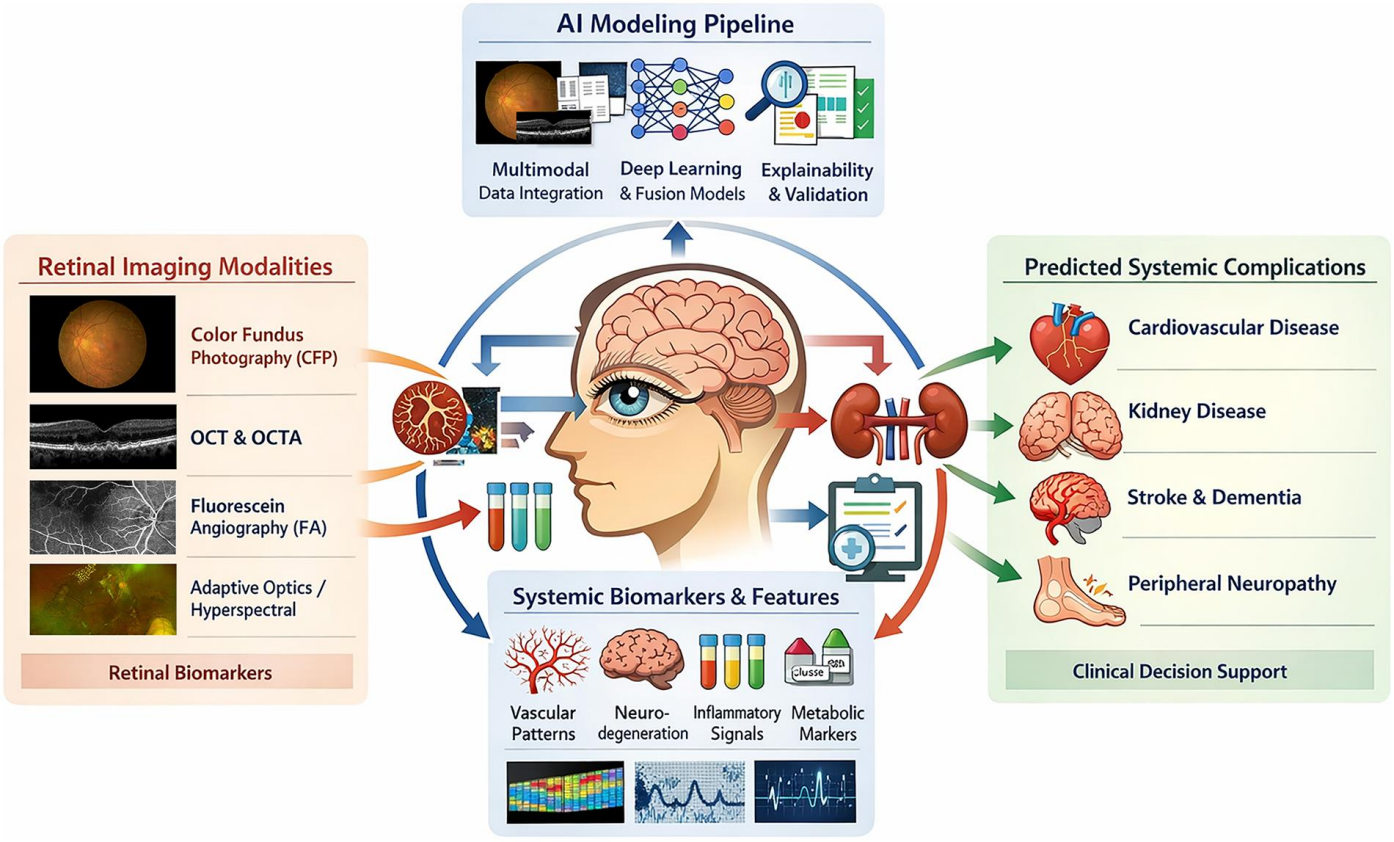
AI-based retinal imaging for systemic risk prediction in diabetes.

To illustrate the overall analytical framework underlying AI-based retinal imaging for systemic complication prediction in T2DM, Figure 2 summarizes the typical pipeline from retinal image acquisition to clinical risk prediction.

### 3.1 Study selection and characteristics

**Figure 3:**
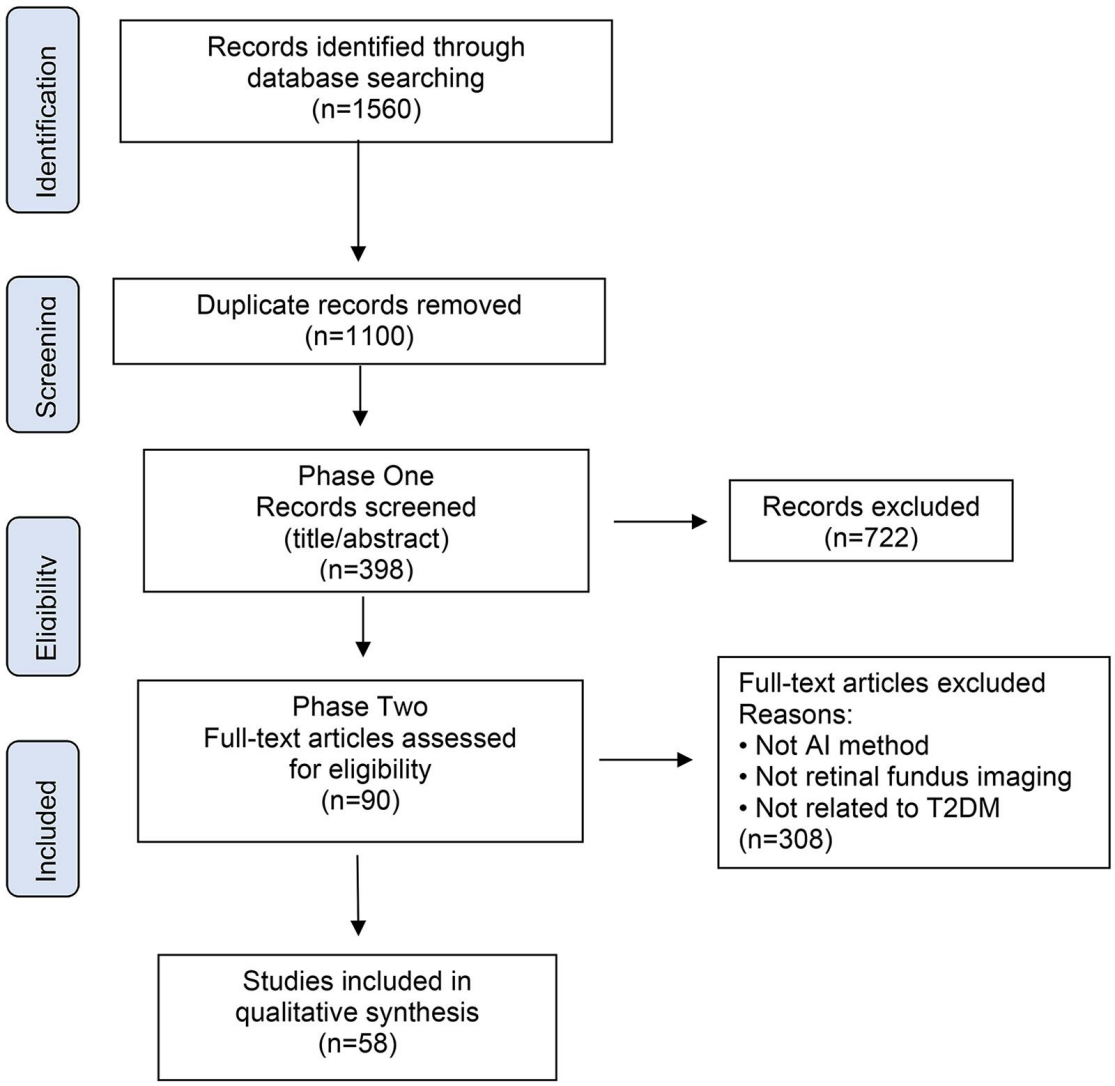
PRISMA-ScR framework.

A total of 58 studies met the predefined eligibility criteria and were included in the final synthesis (Figure 3). The included studies demonstrated substantial heterogeneity in geographic distribution, cohort size, imaging modality, modeling strategy, and validation design. Sample sizes ranged from small proof-of-concept datasets to large-scale population cohorts exceeding tens of thousands of participants, reflecting both exploratory and implementation-oriented research stages.

Imaging modalities varied considerably and included conventional CFP, ultra-widefield (UWF) imaging, OCT, and OCT angiography OCTA. Most studies relied primarily on CFP due to its accessibility and scalability, whereas OCT and OCTA were more frequently used in studies investigating neurodegenerative or microvascular biomarkers. Regarding modeling strategies, deep learning approaches—predominantly convolutional neural networks (CNNs) and, in recent studies, Vision Transformers (ViTs)—were most commonly adopted. A smaller subset employed traditional machine learning models such as Random Forest, LASSO regression, and XGBoost. Several studies developed multimodal frameworks integrating retinal imaging with demographic variables, biochemical markers, or electronic health records (EHRs), reflecting a shift toward clinically integrated prediction systems.

Validation strategies were inconsistent across studies. Internal random splits and k-fold cross-validation were frequently used, while true external validation on geographically independent cohorts was performed in only a minority of publications. Although discrimination metrics such as area under the receiver operating characteristic curve (AUC) were widely reported, calibration assessment, decision curve analysis, and subgroup evaluation were inconsistently described.

### 3.2 Outcomes

Across the included literature, AI-based retinal models were developed to predict diverse systemic outcomes in T2DM. These outcomes clustered into five principal domains: cardiovascular, cerebrovascular, renal, neurological and multi-oragan complications. Table 1 summarizes representative studies that applied retinal imaging–based AI models to predict systemic complications in T2DM.

**Table 1:** Representative AI applications for systemic complication prediction from retinal images in T2DM.

Target complication	Retinal biomarkers used	AI methodology	Data inputs	Reported performance (AUC)	Clinical implication
CVD and Hypertension (4, 15–18)	DR severity, vascular caliber, vessel tortuosity, fractal dimension	CNN-based deep learning models; multimodal fusion networks	Fundus images + clinical risk factors	∼0.78–0.87	Early cardiovascular risk stratification using non-invasive retinal screening
DKD and Microalbuminuria (MAU) (3, 12, 19, 20)	Microvascular lesions, vessel tortuosity, reduced fractal dimension	ML models (XGBoost, Random Forest) and DL frameworks	Retinal features + laboratory biomarkers (HbA1c, inflammatory markers)	Up to ∼0.87	Prediction of microalbuminuria and CKD progression
Cerebrovascular Disease/Stroke (21)	Retinal vascular geometry, arteriolar narrowing, venular dilation	CNN and multimodal predictive models	Fundus images ± clinical variables	∼0.8–0.9	Identification of stroke risk and vascular dysfunction
Neurological Complications (22, 23)	RNFL thinning, GCC thickness reduction	Deep learning models analyzing OCT images	OCT imaging data	Variable; moderate-to-high discrimination	Detection of neurodegenerative and neuropathic processes
Multi-oragan risk prediction (24)	Combined vascular, structural, and neurodegenerative retinal biomarkers	Multimodal AI integrating retinal imaging with EHR and biochemical data	Fundus + OCT + clinical datasets	∼0.78–0.90 depending on outcome	Comprehensive systemic risk modeling in diabetes

These studies collectively highlight the potential of retinal biomarkers as non-invasive indicators of systemic microvascular disease. Among these outcome domains, cardiovascular complications have received the greatest research attention due to their high prevalence and mortality burden in T2DM. Cardiovascular outcomes represented the most frequently investigated category. Large-scale clinical evidence demonstrates a graded association between DR severity and future cardiovascular events. Minimal non-proliferative DR (NPDR) significantly increases the hazard of myocardial infarction (MI), congestive heart failure (CHF), and all-cause mortality (HR 1.15–1.31), with risks escalating substantially in moderate-to-severe NPDR (HR 1.55–1.92) and proliferative DR (PDR) (HR 1.87–2.53) (17). Severe stages such as PDR and diabetic macular edema (DME) are particularly associated with incident and fatal cardiovascular disease. Moreover, DR progression dynamics themselves may serve as early indicators of systemic microvascular vulnerability, as even mild NPDR can demonstrate rapid short-term progression (25). These findings provide biological plausibility for AI-based cardiovascular risk prediction using retinal biomarkers.

Beyond overt DR staging, quantitative vascular geometry metrics, including arteriolar narrowing, venular dilation, tortuosity and reduced fractal dimension, reflect systemic vascular remodeling and microvascular rarefaction. Prospective studies have linked these parameters to hypertension, incident cardiovascular disease, stroke risk, and diabetic nephropathy (18). Reduced fractal dimension was independently associated with incident DR (OR = 0.75; 95% CI: 0.58–0.96) (18), reinforcing the concept of shared systemic microvascular pathophysiology.

Renal complications constituted the second major domain. Retinal microvascular lesions, including intraretinal hemorrhages and vascular remodeling patterns, correlate with DKD severity. AI operationalization of these features has yielded promising results. Machine learning models such as XGBoost (AUC = 0.803) (20), and Random Forest (AUC = 0.87 for microalbuminuria) (19) demonstrated strong predictive performance when combining retinal and systemic variables. These findings position the retina as a non-invasive surrogate of renal microvasculopathy.

Neurological and cerebrovascular complications represent an emerging frontier. RNFL thinning measured by OCT correlates with peripheral and autonomic neuropathy (23), suggesting shared neurodegenerative pathways. Additionally, ML-based analysis of fundus images has demonstrated high discrimination between ischemic and hemorrhagic stroke (AUC 0.929 and 0.951, respectively) (21), highlighting the retina's potential for non-invasive cerebrovascular triage. Beyond retinal imaging, AI applied to other vascular beds also shows promise. A study using a carotid ultrasound-based radiomics nomogram, which integrated imaging features with clinical factors like the triglyceride-glucose index, effectively predicted ischemic stroke risk in T2DM patients, achieving an AUC of 0.898, thereby offering another convenient and customizable tool for cerebrovascular risk stratification (26). The foundational work by Kermany's study (27) demonstrated the power of deep learning not only in classifying retinal diseases but also in providing interpretable diagnoses by highlighting regions of interest, a concept that is now being extended to systemic risk prediction. Kermany's study Similarly, the breadth of AI application in ophthalmology has expanded significantly, with recent efforts like the RFMiD challenge focusing on detecting a wide array of both frequent and rare retinal pathologies, which is crucial for developing generalizable screening models for broader populations (27, 28).

### 3.3 Model architectures and imaging modalities

AI methodologies for retinal analysis have evolved from traditional feature-engineered pipelines to deep representation learning and multimodal fusion systems. Early machine learning approaches relied on handcrafted vascular features followed by classifiers such as support vector machines or Random Forests (29, 30). While interpretable, these models were limited by feature design constraints and demonstrated plateaued performance for complex systemic risk prediction tasks (20, 31). The major stages in the evolution of artificial intelligence architectures applied to retinal image analysis are summarized in Table 2.

**Table 2:** Evolution of artificial intelligence architectures in retinal image analysis.

Dimension	Early ML era	DL & hybrid models	Multi-modal fusion systems	Emerging AI solutions
Modeling architectures	Two-step pipelines: handcrafted features + classifiers (SVM, Random Forest, logistic regression)	End-to-end CNN architectures (ResNet, DenseNet) and Vision Transformers; hybrid CNN-RNN models for temporal imaging	Modality-specific subnetworks integrated via early, late, or intermediate fusion	Federated learning, domain adaptation, privacy-preserving AI
Feature representation	Expert-defined vascular metrics and lesion features	Hierarchical features automatically extracted from raw pixels	Joint feature spaces integrating retinal images with clinical variables, biochemical markers, and EHR data	Self-supervised learning and foundation models trained on large unlabeled datasets
Predictive capability	Adequate for DR detection but limitied for systemic risk prediction	High performance for DR grading and lesion detection; expert-level accuracy in controlled datasets	Improved prediction of cardiovascular, renal, and neurological complications	Robust generalization across populations and imaging devices
Clinical applications	DR screening and lesion identification	DR severity grading, macular edema detection, early complication prediction	Multi-organ risk prediction (CVD, DKD, neuropathy)	Personalized risk stratification and longitudinal monitoring
Key limitations	Dependence on manual feature engineering	Limited generalizability across institutions and imaging devices	Data integration complexity and heterogeneous annotations	Need for large-scale prospective validation

The advent of deep learning enabled end-to-end feature extraction directly from pixel-level data. Prior work has demonstrated the feasibility of interpretable deep learning in retinal image analysis (27). The subsequent rapid growth in this area is underscored by comprehensive reviews like that of Yao's study, which detail the latest AI-driven methodologies for diabetic retinopathy and diabetic macular edema, encompassing disease identification, patient profiling, and prediction of disease progression (32). Furthermore, the creation and utilization of diverse datasets, such as the “Retinal Fundus Multi-disease Image Dataset” (RFMiD) used in the challenge described, are critical for training robust models capable of detecting a wide spectrum of pathologies, moving beyond the detection of only the most common diseases (27, 28, 32). Convolutional neural networks such as ResNet and DenseNet, and more recently Vision Transformers, achieved expert-level performance in DR grading and lesion segmentation (22, 33, 34). The utility of Vision Transformers extends beyond retinal imaging; for instance, a ViT-based model has demonstrated superior performance over standard CNNs in diagnosing coronary layered plaque from optical coherence tomography (OCT) images, achieving an AUC of 0.886, highlighting the versatility and power of this architecture for cardiovascular risk stratification (35). Hybrid architectures integrating CNNs with recurrent neural networks further leveraged temporal information in sequential retinal scans, improving progression modeling (34). Optimized CNN variants incorporating adaptive filters and metaheuristic optimization achieved accuracies approaching 98% in controlled settings (36).

Multimodal fusion strategies represent a key methodological advancement. These architectures combine retinal imaging with clinical variables, biochemical markers (e.g., HbA1c, LDL-C), and EHR-derived endpoints. Early fusion, late fusion, and intermediate feature fusion approaches have been implemented, with transformer-based cross-modal attention mechanisms improving flexibility and performance (37, 38). Multimodal systems have demonstrated additive predictive value for cardiovascular and renal outcomes, increasing AUC for major adverse cardiovascular events prediction from 0.697 to 0.728 when combining retinal, clinical, and polygenic risk scores (16).

Imaging modality selection influences model capability. CFP remains dominant due to scalability, whereas OCT provides quantitative neurostructural metrics such as RNFL and GCC thickness, and OCTA enables capillary-level microvascular mapping (39–41). However, device variability and acquisition heterogeneity introduce domain shift challenges, which remain insufficiently addressed in many studies.The major retinal imaging modalities used for AI-based systemic risk prediction and their respective advantages and limitations are summarized in Table 3.

**Table 3:** Retinal imaging modalities and their role in AI-based systemic risk prediction in T2DM.

Imagind modality	Structural/functional information	Typical AI-extracted features	Advantages for AI modeling	Limitations	Relevant systemic associations/references
CFP	2D imaging of retinal vasculature and lesions; includes microaneurysms, hemorrhages, exudates; UWF extends peripheral view	Vessel caliber, tortuosity, fractal dimension, lesion counts, DR severity grading	High accessibility; suitable for large-scale screening;large datasets available for AI training	Limited depth information; limited functional or perfusion data	Cardiovascular risk prediction; hypertension prediction; systemic vascular health indicators (18, 24, 40)
Optical Coherence Tomography (OCT)	Cross-sectional imaging of retinal layers; quantifies RNFL thickness, GCC thickness, macular edema	Layer thickness metrics, volumetric structural features, neurodegenerative markers	High spatial resolution; quantitative metrics suitable for deep learning feature extraction	Limited field of view; equipment cost; requires trained acquisition	Neuropathy, neurodegeneration, cognitive decline associations (23, 39, 42)
OCT Angiography (OCTA)	Non-invasive microvasculature and capillary networks	FAZ area, vessel density, perfusion metrics, microvascular dropout	Sensitive to early microvascular alterations; strong biomarker potential for systemic microangiopathy	Motion artifacts; signal attenuation in media opacity	Diabetic nephropathy risk, microvascular disease, cardiovascular risk (42, 43)
Fluorescein/ICG Angiography (FA/ICGA)	Dye-based imaging of retinal and choroidal vasculature	Perfusion patterns, leakage detection, vascular remodeling	Functional gold standard for vascular pathology	Invasiveness; risk of adverse reactions; unsuitable for large-scale AI screening	Historically used in vascular disease characterization; increasingly replaced by OCTA (44)
Emerging Modalities (AOSLO, Hyperspectral)	Cellular-level imaging (AOSLO) or metabolic/oxygenation mapping (hyperspectral)	Cellular morphology, metabolic signatures, oxygen saturation patterns	Potential detection of earliest metabolic and microvascular alterations	Experimental; limited dataset; high cost	Early metabolic dysfunction and microvascular disease indicators (39, 45)

### 3.4 Methodological quality landscape

Across studies, methodological rigor varied substantially. Internal validation using random splits or cross-validation predominated, whereas external validation on independent cohorts was limited. This raises concerns regarding overfitting and inflated performance estimates.

Calibration assessment, decision curve analysis, and clinical threshold reporting were inconsistently performed, limiting evaluation of clinical utility. Subgroup analyses by age, sex, ethnicity, or imaging device type were rarely reported, precluding assessment of algorithmic fairness. Studies have documented AUC performance gaps exceeding 12% between racial subgroups in DR grading models (46), underscoring the need for systematic bias evaluation.

Explainability techniques such as Grad-CAM were applied in several studies; however, validation of interpretability outputs was seldom conducted. As a result, the clinical reliability of highlighted regions remains uncertain.

## 4 Discussion

### 4.1 Interpretation

This scoping review synthesizes emerging evidence that retinal imaging, when combined with artificial intelligence, extends far beyond diabetic retinopathy detection and increasingly functions as a systemic biomarker platform for multi-organ risk stratification in type 2 diabetes. The biological plausibility of this paradigm is strongly supported by longitudinal epidemiological studies demonstrating graded associations between DR severity and cardiovascular outcomes (17), as well as microvascular geometry alterations predictive of hypertension and incident vascular disease (18). Retinal neurodegenerative markers, including RNFL thinning and ganglion cell complex loss detected via OCT, further correlate with diabetic neuropathy and central neurodegeneration (23, 41), reinforcing the concept of the retina as an accessible window into systemic vascular and neuronal health. These developments illustrate the growing transition of retinal AI from proof-of-concept research toward clinically decision-support systems.

Importantly, several included studies demonstrated that even early-stage retinal microvascular alterations—such as minimal NPDR—carry prognostic implications for myocardial infarction, congestive heart failure, and mortality (17, 25). This aligns with mechanistic insights suggesting that retinal microangiopathy mirrors generalized endothelial dysfunction and capillary rarefaction (18, 39, 40). AI models trained on such microstructural patterns may therefore capture preclinical systemic vulnerability before overt organ failure develops.

However, reported AUC values ranging from 0.73 to 0.97 across cardiovascular, renal, and cerebrovascular predictions should be interpreted cautiously (14, 16, 19–21, 23, 47–49). Discrimination metrics alone do not reflect calibration quality, clinical decision thresholds, or longitudinal stability. Several studies relied exclusively on internal validation or random splits (29, 31, 50, 51), which are known to inflate performance estimates compared to geographically external validation. In addition, some studies may be vulnerable to overfitting due to limited sample sizes relative to the complexity of deep learning architectures. Moreover, heterogeneity in outcome definitions, follow-up duration, and event adjudication limits cross-study comparability. Thus, while the aggregate evidence is promising, it remains predominantly exploratory rather than implementation-ready.

The evolution from handcrafted vascular feature engineering (29, 51) to deep convolutional neural networks and transformer-based architectures (24, 34, 36) has substantially improved representational capacity. Multimodal fusion approaches integrating retinal imaging with clinical biomarkers and EHR-derived variables (37, 38) further enhanced predictive accuracy for major adverse cardiovascular events and renal decline (16, 49). These findings suggest that retinal data provide complementary rather than standalone predictive value within broader risk modeling ecosystems. The paradigm of using retinal imaging for systemic health forecasting is powerfully articulated, whose review highlights the substantial potential of AI-based retinal biomarkers in predicting neurodegenerative, cardiovascular, and chronic kidney diseases. They emphasize that the retina's unique features enable a non-invasive window into the central nervous system and microvascular circulation, a concept that is central to the studies reviewed in our paper (52).

### 4.2 Explainability

Explainability remains central to clinical acceptance and regulatory integration of AI-based retinal systems. Visualization techniques such as Grad-CAM have consistently highlighted perivascular and optic disc regions when predicting hypertension, cardiovascular risk, and systemic metabolic status (4, 15). These saliency distributions are biologically coherent, given the established relationship between vascular caliber, tortuosity, and systemic hemodynamics (18, 39).

Nevertheless, visualization-based interpretability provides associative rather than causal explanations. Several studies applying CNN and hybrid architectures (22, 33, 34, 36) did not formally validate whether highlighted regions correspond to clinically meaningful microvascular lesions. Furthermore, attention maps may be unstable under minor perturbations, raising concerns regarding explanation reliability. Future work should incorporate quantitative interpretability metrics, counterfactual testing, and clinician-blinded validation experiments to ensure that explanations reflect pathophysiologically relevant structures rather than dataset artifacts. The importance of model interpretability, as demonstrated in the early work, cannot be overstated. Their framework provided a more transparent diagnosis by highlighting the regions recognized by the neural network, a practice that remains essential for building clinical trust and validating that models are focusing on pathophysiologically relevant structures (27).

In addition, as multimodal transformer frameworks become more prevalent (37, 38), explainability must expand beyond pixel-level saliency toward cross-modal attention transparency, particularly when integrating biochemical or genomic data (16). Without such transparency, model decisions risk becoming increasingly opaque as architectural complexity grows.

### 4.3 Domain shift

Domain shift represents one of the most significant translational barriers identified across included studies. Performance degradation has been observed when algorithms trained on homogeneous datasets are applied to ethnically diverse or geographically distinct populations (15, 46). For example, subgroup analyses in DR detection models demonstrated AUC differences exceeding 12% between racial groups (46), raising concerns about algorithmic fairness and systemic bias.

Device heterogeneity further compounds this issue. Variations in camera manufacturer, field-of-view, illumination conditions, and image resolution can alter pixel-level distributions, challenging deep model generalization (22, 33). OCT and OCTA devices introduce additional inter-manufacturer variability in layer segmentation and vascular density quantification (39–41). Few studies implemented structured domain adaptation, adversarial training, or federated learning frameworks to mitigate these effects (37, 38).

Although generative augmentation and transfer learning approaches have shown theoretical promise (36, 38), standardized benchmarking across independent external cohorts remains rare (14, 16, 47–49). Regulatory approval pathways increasingly demand evidence of consistent performance across demographic strata and device types (53–55). Therefore, future research must prioritize multi-center prospective validation, fairness auditing, and recalibration protocols before large-scale deployment.

### 4.4 Clinical translation

While our review notes the promising performance of predictive models, reviews like Yao's study caution that there is often a disparity between the potential of AI models and their actual effectiveness in real-world clinical applications (32). Overcoming challenges in deployment, regulatory compliance, and seamless integration into existing healthcare workflows is essential for these technologies to realize their full potential (32). The development of comprehensive, multi-disease datasets, as championed by the RFMiD challenge described, is a crucial step toward building generalizable models that can be reliably deployed in diverse clinical settings, moving beyond the proof-of-concept stage toward implementation-ready tools (28). The ultimate goal is to leverage AI-based retinal imaging to transform primary care and systemic disease management through early detection and personalized care plans, a vision that underscores the translational urgency highlighted in our conclusion (52).

From a translational perspective, AI-enabled retinal assessment aligns conceptually with the movement toward integrated diabetes care and early complication surveillance. FDA-cleared autonomous DR systems (53, 55) demonstrate that regulatory pathways for retinal AI are feasible. For instance, a deep learning model using Inception V3 architecture achieved high diagnostic performance for severe DR (AUC 0.968), demonstrating the technical capability of AI to accurately identify vision-threatening conditions, thereby potentially increasing the accessibility and efficiency of large-scale screening programs (56). However, extending from disease detection to multi-organ prognostication substantially increases evidentiary requirements.

Most predictive models for cardiovascular and renal outcomes remain retrospective and observational (14, 16, 47–49). Prospective impact studies evaluating whether AI-informed screening alters therapeutic decision-making, improves risk factor control, or reduces event rates are lacking. Cost-effectiveness analyses are similarly underreported, despite the high global burden of T2DM-related complications (57, 58).

Clinical workflow integration also warrants consideration. AI outputs must be interpretable, threshold-calibrated, and seamlessly embedded within EHR systems to support endocrinologists, cardiologists, and primary care physicians. Furthermore, medicolegal accountability frameworks remain underdeveloped (54, 59, 60). Liability distribution between developers, healthcare institutions, and clinicians requires regulatory clarification to facilitate responsible adoption. Critically, the successful translation of AI into practice hinges not only on technical and regulatory readiness but also on the perceptions and acceptance of its end-users. A recent survey within the English NHS Diabetic Eye Screening Programme found that both people living with diabetes and healthcare practitioners believe AI integration is inevitable and could improve efficiency. However, concerns about job losses, data security, and the safety of AI-driven decisions were prevalent, alongside misconceptions about AI's role in patient interactions. This underscores the urgent need for targeted education to build trust and facilitate the smooth integration of AI into screening workflows (61).

Importantly, retinal AI should be conceptualized as an augmentation tool rather than a replacement for clinical expertise. Hybrid decision-support systems combining AI risk estimates with clinician judgment may offer the most pragmatic implementation pathway. Continuous monitoring, periodic model recalibration, and performance auditing will be essential to maintain safety and effectiveness over time (16, 30, 54, 62, 63).

## 5 Conclusion

This review underscores the retina as a non-invasive biomarker hub reflecting systemic microvascular and neurodegenerative processes in T2DM. AI models leveraging retinal imaging—particularly multimodal deep learning systems—demonstrate promising performance for predicting cardiovascular, renal, cerebrovascular, and neurological complications.

However, translation into routine care requires rigorous external validation, standardized reporting, fairness evaluation, domain-shift mitigation, and prospective outcome studies. With coordinated efforts across ophthalmology, endocrinology, data science, and regulatory governance, AI-enabled retinal assessment may evolve into a scalable platform for integrated, preemptive, and personalized multi-organ risk management in type 2 diabetes.
